# Differential gene expression profile in pig adipose tissue treated with/without clenbuterol

**DOI:** 10.1186/1471-2164-8-433

**Published:** 2007-11-26

**Authors:** Jin Zhang, Qiang He, Qiu Y Liu, Wei Guo, Xue M Deng, Wei W Zhang, Xiao X Hu, Ning Li

**Affiliations:** 1 State Key Laboratory for Agrobiotechnology, China Agricultural University, Beijing 100094, China; 2 Life Science and Biotechnology Department, HeBei Normal University of Science & Technology, Qinhuangdao, 066600, China; 3 College of Animal Science and Technology, China Agricultural University, Beijing 100094, China

## Abstract

**Background:**

Clenbuterol, a beta-agonist, can dramatically reduce pig adipose accumulation at high dosages. However, it has been banned in pig production because people who eat pig products treated with clenbuterol can be poisoned by the clenbuterol residues. To understand the molecular mechanism for this fat reduction, cDNA microarray, real-time PCR, two-dimensional electrophoresis and mass spectra were used to study the differential gene expression profiles of pig adipose tissues treated with/without clenbuterol. The objective of this research is to identify novel genes and physiological pathways that potentially facilitate clenbuterol induced reduction of adipose accumulation.

**Results:**

Clenbuterol was found to improve the lean meat percentage about 10 percent (P < 0.05). The adipose cells became smaller and the muscle fibers became thicker with the administration of clenbuterol. The mRNA abundance levels of 82 genes (ESTs) were found to be statistically differentially expressed based on the Student t-test (P < 0.05) in the microarray analyses which contained 3358 genes (ESTs). These 82 genes (ESTs) were divided into four groups according to their Gene Ontology Biological Process descriptions. 16 genes were cellular metabolism related genes (including five related to lipid metabolism such as apolipoprotein D and apolipoprotein R), 10 were signal transduction related genes, 45 were expressed sequence tags (ESTs) and 11 others were of various categories. Eleven of the 82 genes (ESTs) were chosen for real-time PCR analysis, with eight genes showing similar induction magnitude as that seen in the microarray data. Apolipoprotein R was also found to be up-regulated by the proteomic analysis.

**Conclusion:**

Pig fat accumulation was reduced dramatically with clenbuterol treatment. Histological sections and global evaluation of gene expression after administration of clenbuterol in pigs identified profound changes in adipose cells. With clenbuterol stimulation, adipose cell volumes decreased and their gene expression profile changed, which indicate some metabolism processes have been also altered. Although the biological functions of the differentially expressed genes are not completely known, higher expressions of these molecules in adipose tissue might contribute to the reduction of fat accumulation. Among these genes, five lipid metabolism related genes were of special interest for further study, including apoD and apoR. The apoR expression was increased at both the RNA and protein levels. The apoR may be one of the critical molecules through which clenbuterol reduces fat accumulation.

## Background

The β_2_-agonist clenbuterol (0.8–3.2 μg/kg body weight twice daily) is used as a bronchodilator for the treatment of asthma in humans and as a bronchodilator as well as a tocolytic agent in veterinary medicine [1]. In the past decade, high dosages of clenbuterol (ten to one hundred times the clinically active dose) have been fed to livestock to improve feed conversion, reduce body fat and increase muscle mass [2,3]. However, people who eat the animal products can be poisoned by the clenbuterol residues [4-6]. Therefore, the use of clenbuterol for growth promotion in food-producing animals is not approved within China, the European community, the United States, and most other countries [2,7].

Clenbuterol influences cell metabolism by combining with β_2_-adrenergic receptors and by increasing the cAMP concentration in cells. In adipocytes, stimulation of β-adrenergic receptors (by hormones) increases cyclic AMP levels and activates protein kinase A (PKA), which stimulates lipolysis by phosphorylating hormone-sensitive lipase and perilipin [8-11]. However the molecular level mechanism by which clenbuterol influences adipose accumulation is still not understood.

Recently, global gene/protein expression analysis techniques using DNA microarray/2-D gel analyses have been widely used to define the characteristics and specific patterns of gene expressions elicited by various toxicants [12-14]. In this study, the molecular level mechanism by which clenbuterol reduces fat accumulation was studied with cDNA microarray and proteomics techniques to analyze the fat tissue of Chinese miniature pigs treated with/without clenbuterol.

## Results

### Adipose accumulation decreased dramatically by clenbuterol administration

HPLC analyses of blood samples showed that the clenbuterol concentrations in the test pigs fed with clenbuterol were about 20 ng/ml in the 3 month-old pigs and about 100 ng/ml in the 4 month-old pigs. Clenbuterol could not be detected in the control pigs fed without clenbuterol (Additional file 1 Table S1). The test pigs and control pigs did not exhibit different weights (Additional file 1 Table S2), but did exhibit different body compositions (Table 1). The effect of clenbuterol on body composition became more dramatic with advancing age. In the 3 month-old group, the lean meat percentage was increased by 2%, the back fat thickness was reduced by ~0.2 cm, and the loin muscle area was reduced by 4.7 cm^2^. This difference was statistically significant (P < 0.05). In the 4 month-old group, the lean meat percentage was increased by 10.99%, the back fat thickness was reduced by 0.38 cm and the loin muscle area was reduced by 2.18 cm^2^. The changes of the lean meat percentage and the loin muscle area were strongly statistically significant (P < 0.01) while the changes of the back fat thickness were statistically significant (P < 0.05). These data indicate that clenbuterol plays a role in pig adipose reduction. The sample quality was sufficient for further analysis to identify molecules with changed expression levels and molecules which impact adipose accumulation.

**Table 1:** Body composition of pigs treated with/without clenbuterol

Age	3 month-old		3 month-old		4 month-old		4 month-old	
Clenbuterol dosage*	0		25		0		50	

Pig	Hog 2	Sow 2	Hog 1	Sow 1	Hog 4	Sow 4	Hog 3	Sow 3
Lean meat percentage of carcass (%)	52.34	52.46	55.09	55.39	41.38	41.52	52.36	52.52
Thickness of back fat (cm)	2.361	2.311	2.132	2.104	3.229	3.171	2.824	2.812
Loin muscle area (cm^2^)	19.460	19.516	24.086	24.312	23.063	23.103	25.228	25.312

*twice daily with the unit of (mg/kg body weight)

The carcass lean meat percentage, the back fat thickness and the loin muscle area were all changed statistically in both the 3 month-old group and the 4 month-old group after clenbuterol treatment (P < .05).

### Muscle fibers became thicker and adipose cells became smaller with the administration of clenbuterol

**Figure 1:**
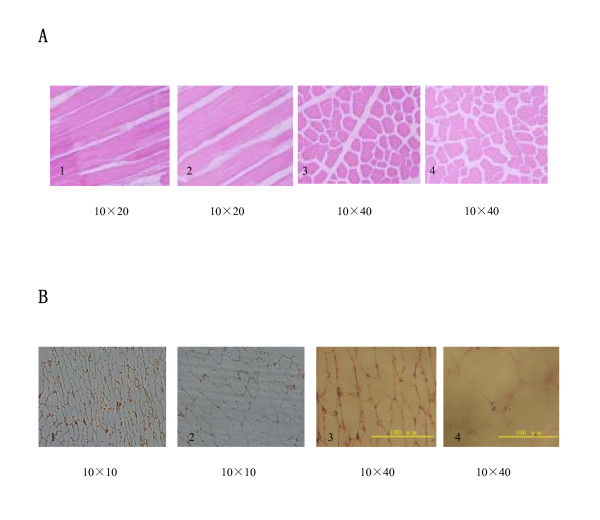
**1A. Skeleton muscle (biceps femoris) histological section of pigs with/without the administration of clenbuterol**. 1. transverse, test pigs. 2. transverse, control pigs. 3. longitudinal, test pigs. 4. longitudinal, control pigs. Vertical fiber sections analyzed with TD2000 real-color pathology image analysis system (Beijing Tiandibainian Scientific Company Ltd.). The muscle fibers of pigs become thicker when treated with clenbuterol. **1B. Subcutaneous back fat (at the fifth lumbar vertebra level) histological section of pigs with/without the administration of clenbuterol**. 1. amplified10 × 10 of test pigs. 2. amplified 10 × 10 of control pigs. 3. amplified 10 × 40 of test pigs. 4. amplified 10 × 40 of control pigs. The sizes of the pig adipose cells decreased when treated with clenbuterol. The cell size was analyzed by counting cells on the slide visible through the microscope eyepiece.

Clenbuterol produces specific protein anabolic effects in skeletal muscle in addition to lipolysis in adipose tissue of various vertebrates [15-18]. However, there has not yet been any research explaining how these two types of tissues are changed by the administration of clenbuterol. Muscle and adipose histological sections were analyzed to understand how these two tissue types are changed (Figure 1A &1B).

Paraffin histological section analyses were done using samples from the 4 month-old pig's biceps femoris. Four histological section slides were prepared for each pig, two transverse muscle fiber slides and two longitudinal muscle fiber slides, for analysis of the cross-sectional area of the fibers with a TD2000 real-color pathology image analysis system (Beijing Tiandibainian Scientific Company, Ltd.). The cross-sectional areas of the muscle fibers of the test pigs and the control pigs were significantly different with a student t-test P value of 0.0224 (Additional file 1 Table S3). The results show that the administration of clenbuterol increased the muscle fiber thicknesses in the pigs.

The 4 month-old pig's back fat tissue (at the fifth lumbar vertebra level) was also analyzed using paraffin histological section. Five random areas on the slides were chosen for the adipose cell size analyses. The cell sizes were analyzed by counting all the cells on the slides visible through the microscope eyepiece at the 10 × 40 magnification (Additional file 1 Table S4). Numbers of cells in the test group were strongly statistically more than in the control group (P < 0.01). Thus, clenbuterol caused a reduction in adipocyte size.

The clenbuterol thickened the pig muscle fibers and reduced the sizes of the pig adipocyte cells in the back fat tissues. Clenbuterol is known to increase muscle mass and reduce body fat. We suggest that clenbuterol increases the muscle mass by thickening the muscle fibers and reduces the body fat by shrinking the adipose cells. The size of the adipose cells depends on the sizes of the lipid droplets in the cells. Therefore, the adipose cells become smaller as the clenbuterol reduces the lipid droplets in the adipose cells.

### cDNA microarray identified 82 genes with changed mRNA abundance in adipose tissue with stimulation by clenbuterol

**Figure 2:**
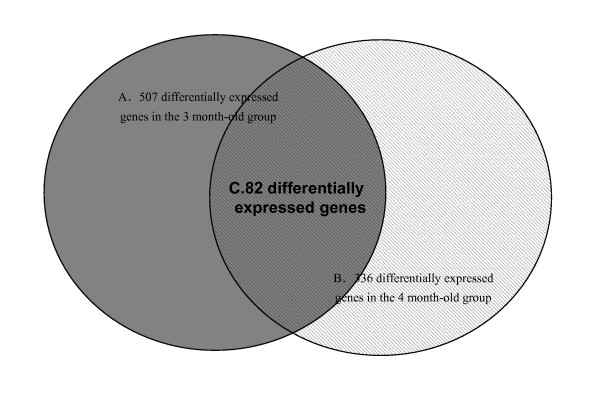
**82 genes were differentially expressed in the DNA microarray analysis in pig fat tissue with clenbuterol administration (P < .05) in both groups**. A. The black circle indicates the 507 differentially expressed genes in the 3 month-old group (P < .05 in four microarray slides). B. The cross-hatched circle indicates the 336 differentially expressed genes in the 4 month-old group (P < .05 in four microarray slides). C. The overlapping region indicates the 82 differentially expressed genes in both the 3 month old group and the 4 month old group that were of interest.

Eight microarray slides were used (four for the 3 month-old group and four for the 4 month-old group) for global evaluation of the gene expression in the adipose tissue after administration of clenbuterol. 8335 spots representing 2770 genes (ESTs) in the 3 month-old group and 8740 spots representing 2862 genes (ESTs) in the 4 month-old group passed the spots quality filter and were analyzed with the Student t test. 507 genes in the 3 month-old group (P < 0.05 in four microarray slides) and 336 genes in the 4 month-old group (P < 0.05 in four microarray slides) were differentially expressed (data not shown). The goal of this study was to identify gene expression profiles affected by clenbuterol. Therefore, genes that were differentially expressed in both groups (82 genes in total) were selected for further study as being differentially expressed in adipose tissue with stimulation by clenbuterol (Fig. 2).

These 82 genes (ESTs) were divided into 4 groups according to their Gene Ontology Biological Process descriptions. 16 genes were cellular metabolism related genes (Table 2), 10 were signal transduction related genes (Table 3), 45 were expressed sequence tags (ESTs) (Table 4) (no homologous sequences were found in the NCBI nucleotide database for 17 ESTs, while the other 28 ESTs hit only some EST sequences without any functional annotations) and the other 11 genes were of various categories (Table 5). 45 EST sequences were deposited at dbEST of NCBI [19].

**Table 2:** Group I of differentially expressed genes: Cell metabolism

**GenBank Access No. (Clone No. on Microarray)**	**Gene name**	Annotation*	**Induction Fold change 3 month & 4 month**	
NM_001647 (rpfat_18926)	Apolipprotein D (ApoD)	Lipid metabolism	1.76	1.67
L06820(rpfat_18262)	Apolipprotein R (ApoR)	Lipid metabolism	2.58	1.80
XM_854434.1(rpig_3250)	Canis familiaris similar to phosphatidic acid phosphatase type 2A isoform 1, transcript variant 2	Lipid metabolism	1.27	1.14
NM_006022 (rpigfat_10082)	transforming growth factor beta-stimulated protein	modulates the frequency, rate or extent of DNA-dependent transcription	1.25	1.13
NM_007158 (rpfat_18317)	NRAS-related gene (D1S155E)		1.25	1.54
NM_014828 (rpfat_16693)	KIAA0737 gene product (KIAA0737)		1.28	1.43
NM_003069(rpfat_18518)	SWI/SNF related, matrix associated, actin dependent regulator of chromatin, subfamily a, member 1 (SMARCA1)		1.23	1.64
NM_000985 (rpfat_19876)	ribosomal protein L17 (RPL17), mRNA	protein translation	1.47	1.09
LOC484530(rpfat_15686)	ribosomal protein S10	protein translation	1.33	1.15
M64620 (rpfat_4179)	cathepsin B	lysosomal cysteine proteinase	1.14	1.13
DQ673096(rpfat_19771)	Eukaryotic translation elongation factor 1 alpha (EEF1A)	protein translation	1.70	1.29
X81197(rpfat_13772)	archain 1	endoplasmic reticulum to Golgi transport	2.15	1.62
AF027652 (rpfat_19670)	L-3-hydroxyacyl-CoA dehydrogenase precursor (HAD) mRNA,	Lipid metabolism	-1.30	1.11
AY487830 (rpfat_17395)	Sus scrofa stearoyl-CoA desaturase (SCD) gene, exons 1 through 6 and complete cds	Lipid metabolism	-1.89	1.03
DQ629164 (rpfat_4193)	ribosomal protein L10a	protein translation	-1.01	1.03
NP_001001636 (rpfat_18915)	ribosomal protein L32	Protein translation	-1.79	-1.01

*according to description of the Gene Ontology Biological Process Category

*All the genes in the table were differentially expressed according to the student t test in the eight microarray slides (P < .05)

**Table 3:** Group II of differentially expressed genes: Signal transduction*

**GenBank Access No. (Clone No. on Microarray)**	**Gene name**	**Annotation**	**Induction Fold change 3 month & 4 month**	
X05942 (rpfat_17661)	cAMP dependent protein kinase type I regulatory (PRKAR1A)	causes the dissociation of the inactive holoenzyme	2.0	2.0
Z33879 (rpfat_17754)	mRNA encoding G-beta like protein (RACK1)	a physiological mediator of agonist-induced Ca^2+ ^release	1.29	1.05
NM_204675.1 (rpfat_17793)	wingless-type MMTV integration site family, member 3A (WNT3A)	leads to an increase in intracellular calcium and activation of protein kinase C (PKC)	1.27	1.09
U57092 (rpfat_19360)	RAB30	member of the RAS oncogene family	2.05	2.41
U05291 (rpfat_10974)	fibromodulin	participate in the assembly of the extracellular matrix as it interacts with type I and type II collagen fibrils	1.28	1.04
M18981 (rpfat_8258)	S100 calcium binding protein A6	helps stimulation Ca^2+^-dependent insulin release, prolactin secretion and exocytosis	5.96	1.61
U01160 (rpfat_8561)	transmembrane 4 superfamily protein (SAS)	growth-related cellular processes	1.09	1.21
AF268463 (rpfat_15672)	voltage-dependent anion channel 3 (VDAC3)	Calcium signaling pathway	-1.26	-1.05
NM_003248 (rpigfat_10263)	thrombospondin 4 (THBS4)	forms a pentamer and can bind to heparin and calcium	-1.04	1.11
BC024040 (rpfat_18349)	Homo sapiens CXXC finger 5, mRNA	up-regulation of I-kappaB kinase/NF-kappaB cascade	-1.41	1.04

*according to description of the Gene Ontology Biological Process Category

*All the genes in the table were differentially expressed according to the student t test in the eight microarray slides (P < .05)

**Table 4:** Group III of differentially expressed genes: Expressed Sequence Tags (ESTs)

**GenBank Access No.**	**Clone No. on Microarray**	**Induction Fold change**	
		
		**3 month**	**4 month**
ES605520	rpfat_9071	1.05	1.06
ES605522	rpig_3811	1.63	1.42
ES605503	rpfat_19982	1.41	1.27
ES605504	rpig_3786	1.01	1.10
ES605505	rpfat_11284	1.21	1.21
ES605524	rpfat_17910	1.55	1.05
ES605516	rpfat_15361	1.58	1.25
ES605525	rpfat_15914	1.38	1.13
ES605526	rpfat_16368	1.55	1.12
ES605506	rpfat_18461	1.33	1.07
ES605507	rpfat_11050	1.41	1.17
ES605508	rpigfat_10350	1.84	1.21
ES605509	rpigfat_10251	1.36	1.21
ES605510	rpig_3845	1.06	1.09
ES605511	rpfat_9552	1.05	1.08
ES605528	rpfat_19923	1.24	1.08
ES605530	rpfat_16045	1.36	1.18
ES605532	rpigfat_10253	1.77	1.26
ES605533	rpig_4030	1.07	1.11
ES605535	rpfat_13525	1.49	1.14
ES605536	rpfat_5467	1.79	1.58
ES605537	rpfat_18469	1.18	1.14
ES605538	rpfat_9125	1.34	1.24
ES605539	rpfat_8471	2.32	1.37
ES605540	rpfat_17820	1.39	1.13
ES605513	rpfat_16623	1.54	1.22
ES605541	rpfat_17845	1.21	1.38
ES605543	rpigfat_10285	1.40	1.08
ES605514	rpfat_18411	1.97	1.57
ES605544	rpfat_12523	1.52	1.33
ES605518	rpig_3750	1.60	1.43
ES605515	rpfat_17081	1.57	1.14
ES605519	rpfat_10949	1.08	1.16
ES652311	rpfat_11494	1.05	1.21
ES652312	rpfat_11443	1.09	1.46
ES652313	rpfat_18636	1.83	1.22
ES605523	rpigfat_9891	-1.28	1.04
ES605512	rpfat_19175	-2	1.00
ES605527	rpfat_18872	-1.28	1.03
ES605529	rpfat_10950	-1.59	1.02
ES605534	rpig_3746	-1.03	1.10
ES605531	rpfat_11274	-1.35	1.01
ES605517	rpfat_12459	-1.10	1.08
ES605542	rpfat_15623	-1.51	1.13
ES605521	rpfat_11483	-1.42	-1.06

*All the genes in the table were differentially expressed according to the student t test in the eight microarray slides (P < .05)

**Table 5:** Group IV of differentially expressed genes: Various categories*

**GenBank Access No. (Clone No. on Microarray)**	**Gene name**	**Annotation**	**Change test/control 3 month & 4 month**	
AF246221 (rpfat_12517)	transmembrane protein BRI	developmental processes	1.27	1.13
AF178980 (rpigfat_9892)	D-prohibitin mRNA	developmental processes	1.54	1.24
NM_000089 (rpfat_12612)	collagen, type I, alpha 2 (COL1A2)	developmental processes	2.48	1.53
Z74616 (rpfat_16033)	mRNA encoding Pro-alpha-2 chain of type I procollagen (COL1A2)	cell structure and mobility	4.20	2.52
AB033007 (rpfat_15395)	mRNA for KIAA1181 protein	cellular localization	1.29	1.08
X06700 (rpfat_18309)	mRNA 3' region for pro-alpha1(III) collagen(COL3A1)	cell structure and mobility	3.16	4
Z74615 (rpfat_8523)	mRNA for prepro-alpha1(I) collagen (COL1A1)	cell structure and mobility	2.86	2.86
X14420 (rpfat_19990)	mRNA for pro-alpha-1 type 3 collagen (COL3A1)	cell structure and mobility	3.29	3.33
BC093076 (rpigfat_10250)	Homo sapiens peptidylprolyl isomerase A (cyclophilin A)	protein folding and stabilization	1.28	1.05
NM_006136 (rpfat_18939)	capping protein (actin filament) muscle Z-line, alpha	cell mobility	2.02	1.61
NM_005915 (rpfat_15643)	minichromosome maintenance deficient (mis5, S. pombe) 6	cell cycle	1.75	1.62

*according to description of the Gene Ontology Biological Process Category

*All the genes in the table were differentially expressed according to the student t test in the eight microarray slides (P < .05).

### 73% cDNA microarray results confirmed by real-time PCR

Seven differentially expressed genes (positive genes) were chosen for real-time PCR analysis with the 4 month-old group samples used to validate the microarray data (Table 6). Clone rpfat_18309 and rpfat_19990 representing pro-alpha-1 type 3 collagen were not detected by the real-time PCR. The other five clones, for genes of apoD (apolipoprotein D), PRKAR1A (cAMP dependent protein kinase type I regulatory), COL1A2 (pro-alpha-2 chain of type I procollagen) and COL1A1 (prepro-alpha1(I) collagen), showed a statistically significant increase in mRNA abundance with administration of clenbuterol (P < 0.05).

**Table 6:** Real-time PCR validation of microarray positive results

Gene* **(Clone No. on Microarray)**	Hog 3/hog 4		Sow 3/sow 4	
	
	Change (test/control)	P value	Change (test/control)	P value
ApoD (rpfat_18926)	12.28	0.013	7.49	0.002
PRKAR1A(rpfat_17661)	6.65	0.014	8.18	0.046
COL1A1 (rpfat_8523)	20.08	0.00008	13.47	0.00001
COL1A2 (rpfat_16033)	2.87	0.0010	3.66	0.0078
COL1A2 (rpfat_17393)	3.00	0.0002	2.29	0.0002
COL3A1(rpfat_18309)	Not detected	Not detected	Not detected	Not detected
COL3A1 (rpfat_19990)	Not detected	Not detected	Not detected	Not detected

* The full names of each gene are listed in Tables 2-5.

Five of the seven genes were confirmed by real-time PCR.

The SCD (stearoyl-CoA desaturase) and HSL (hormone-sensitive lipase) genes are very important in lipid metabolism. The mRNA abundance of stearoyl-CoA desaturase (SCD) decreased in the 3 month-old group and increased in the 4 month-old group, while HSL was not significantly differentially expressed by stimulation with clenbuterol in the microarray analysis. PMP22 (peripheral myelin protein 22) and PHPT1 (phosphohistidine phosphatase 1) were also not significantly differentially expressed by stimulation with clenbuterol in the microarray analysis. These four genes (negative genes) were not significantly differentially expressed by the real-time PCR analysis with 8 pig samples (P > 0.05) (Table 7). In total, 11 genes were analyzed by the real-time PCR. Eight genes showed similar induction magnitude as that seen in the microarray data; two could not be detected, and one was inconsistent with the microarray results. Thus, these results provide strong biological validation of the results from the microarray experiment.

**Table 7:** Real-time PCR validation of microarray negative results

Gene* **(Clone No. on Microarray)**	Hog 1/hog 2		Sow 1/sow 2		Hog 3/hog 4		Sow 3/sow 4	
	
	Change (test/control)	P value	Change (test/control)	P value	Change (test/control)	P value	Change (test/control)	P value
SCD (rpfat_16685)	1.56	0.1680	0.77	0.2119	1.49	0.0822	1.23	0.9943
HSL (rpfat_11096)	1.29	0.0679	1.11	0.2989	1.29	0.3462	1.06	0.5294
PMP22^a ^(rpfat_18575)	1.15	0.6198	1.16	0.3178	1.24	0.6900	1.46	0.1175
PHPT1^b ^(rpfat_15312)	0.75	0.1218	0.95	0.5811	1.24	0.4694	1.46	0.4869

* The full names of each gene are listed in Tables 2-5.

a : PMP22: peripheral myelin protein 22

b :PHPT1: Phosphohistidine phosphatase 1

Three genes (HSL, PMP22 and PHPT1) were confirmed by real-time PCR.

### Apolipoprotein R protein highly presented in adipose with the administration of clenbuterol

**Figure 3:**
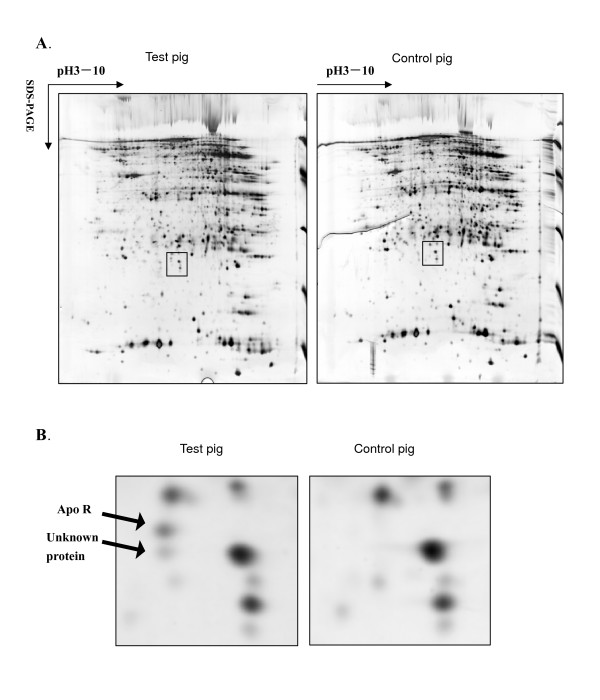
**Proteomic study of adipose tissue of pigs with/without the administration of clenbuterol**. A. Two-dimensional gel analysis of total proteins from adipose tissue treated with/without clenbuterol. (13% SDS-PAGE, silver stain) 600 ± 50 protein spots were detected. B. Two differentially expressed protein spots (Amplified from the frame in A).

After spot detection, background subtraction and volume normalization, 600 ± 50 protein spots were detected in adipose cells using two-dimensional electrophoresis methods. Two spots found to be only expressed in the test group were chosen for digestion in-gel for peptide mass fingerprint (PMF) analysis with a mass spectrograph (Fig. 3A &3B). A Mascot search using the PMF data matched seven of the peptides with peptides from apolipoprotein R, with a sequence coverage of 36% and an expectation of 0.00043 [20]. The other differentially expressed protein did not give any positive results in the database search.

## Discussion

To identify the genes responding to clenbuterol treatment in adipose tissue, a cDNA microarray, real-time PCR and 2-dimensional protein gel analysis were used. 82 genes were identified as being differentially expressed by the microarray analysis with student t test (p < 0.05). These were categorized into to four groups.

### Lipid metabolism related genes in group I

16 differentially expressed genes are involved in cellular metabolism (Table 2). Five of them, including apoD (apolipoproteinD), apoR (apolipoprotein R), HAD (L-3-hydroxyacyl-CoA dehydrogenase precursor), PAP type 2A isoform 1 (phosphatidic acid phosphatase type 2A isoform 1) and SCD (stearoyl-CoA desaturase), directly participate in lipid metabolism.

Both apoD and apoR were up-regulated by the administration of clenbuterol. ApoD is a component of high density lipoproteins [21]. ApoR is a 23-kDa protein found on very low-density lipoproteins (VLDL), on chylomicrons, and in the d > 1.21 g/ml fraction of pig plasma [22]. ApoR was also found to be up-regulated by proteomic analysis. Although the physiologic functions of apoD and apoR in adipose tissue are unknown, they may respond to clenbuterol stimulation to alter lipid metabolism.

Type 2 PAPs appear to metabolize a wide range of lipid mediators derived from both glycero- and sphingolipids [23,24]. SCD is the enzyme responsible for conversion of saturated fatty acids into monounsaturated fatty acids (MUFA) in mammalian adipocytes [25]. HAD is a mitochondrial protein that catalyzes the oxidation of a wide variety of fatty acids, alcohols, and steroids [26,27].

The identification of these molecules is not sufficient to draw out the clenbuterol physiological pathway. However, with the study of the ESTs in Table 4, some new functional genes related to lipid metabolism will be identified. The mechanism of clenbuterol reducing fat accumulation will be revealed on the molecular level based on these differentially expressed genes.

### Other cellular metabolism related genes in group I

Of the other cellular metabolism related genes, EEF1A (eukaryotic translation elongation factor 1 alpha) is responsible for the enzymatic delivery of aminoacyl tRNAs to the ribosome. EEF1A up-regulation indicates that translation activity in adipose cells is enhanced by clenbuterol stimulation. Aarchain 1 may be involved in vesicle structure or trafficking [28] and its up regulation suggests that transport of cargos from the endoplasmic reticulum (ER) to the Golgi was enhanced. Cathepsin B is a lysosomal cysteine proteinase composed of a dimer with disulfide-linked heavy and light chains. D1S155E (NRAS-related gene), SMARCA1 (SWI/SNF related, matrix associated, actin dependent regulator of chromatin, subfamily a, member 1) and KIAA0737 (KIAA0737 gene product) modulate the frequency, rate or extent of DNA-dependent transcription.

Together, these findings indicate that some metabolic processes, such as DNA transcription, protein translation and protein translocation, were enhanced in the adipose cells by clenbuterol stimulation.

### cAMP signaling pathway related genes in group II

The beta-androgenic receptor (β-AR) signal pathway has been well described previously [11]. Clenbuterol as a beta-agonist works by binding with β-AR which then transmits signals into the cell along the G protein-mediated cAMP signal pathway [29]. However, the molecular level mechanisms by which clenbuterol affects adipose accumulation are still unknown. To examine the expression level of genes related to cAMP or G-protein, a total of 11 genes were found directly related to cAMP or G-protein in the microarray gene list. Only two of the eleven were found to be up-regulated, the cAMP dependent protein kinase type I regulatory gene (PRKAR1A) and RAB30 (Table 3). Up-regulation of PRKAR1A was confirmed by the real-time PCR. No reports have been found concerning functions of PRKAR1A and RBA30 in adipose cells.

### Other signal transduction related genes in group II

Another seven signal transduction genes were found to be differentially expressed (Table 3). Five genes (RACK1, WNT3A, VDAC3, S100A6, THBS4) encoding signal transduction molecules are calcium related. This indicates that clenbuterol may have an impact on calcium signaling pathways in cells. Both RACK1 and WNT3A activate protein kinase C, while protein kinase A has previously been reported as the target of clenbuterol. All this data suggests that clenbuterol's effects on adipose cell may be more complex than previously suggested.

Fibromodulin participates in the assembly of the extracellular matrix as it interacts with type I and type II collagen fibrils [30]. Several collagen genes were also found to be up-regulated (Table 5). This indicates that clenbuterol may have an effect on cell collagen synthesis which may alter the cell matrix composition. It is difficult to determine whether this has any relationship to trigalloyl glycerol accumulation.

### ESTs sequence in group IV

45 differentially expressed ESTs are listed in Table 4. Specific conclusions can not be derived from these ESTs results at this time. Some key genes for lipid metabolism, which are unknown at this time, may be found from this list in the future.

### Clenbuterol stimulates genes up-regulated

Only three genes were found to be down-regulated in the 82 differentially expressed genes. To further investigate whether some genes were down-regulated by the administration of clenbuterol, PHPT1 (phosphohistidine phosphatase 1) and PMP22 (peripheral myelin protein 22) were analyzed by real-time PCR (Table 7). These two genes were down-regulated in the 3 month-old group (p = 0.03), but they were not statistically differentially expressed in the 4 month-old group (p = 0.09) by the microarray analysis. The PCR results showed that the mRNA expression of these two genes were not changed significantly (P > 0.05) which confirms the unusual microarray result that very few genes were suppressed. This data suggests that clenbuterol stimulates gene up-regulation in adipose cells.

## Conclusion

Pig fat accumulation was reduced dramatically with clenbuterol treatment. Histological sections and global evaluation of gene expression after administration of clenbuterol in pigs identified profound changes in adipose cells. With clenbuterol stimulation, adipose cell volumes decreased and their gene expression profile changed, which indicate some metabolism processes have been also altered. Although the biological functions of the differentially expressed genes are not completely known, higher expressions of these molecules in adipose tissue might contribute to the reduction of fat accumulation. Among these genes, five lipid metabolism related genes were of special interest for further study, including apoD and apoR. The apoR expression was increased at both the RNA and protein levels. The apoR may be one of the critical molecules through which clenbuterol reduces fat accumulation.

## Methods

### Animal sampling and clenbuterol treatment

Eight Chinese miniature pigs were used in the experiments. Four hogs and four sows, all 4 weeks old, were housed in the Nutrition and Metabolism Laboratory at the China Agriculture University. They were raised under exactly the same conditions and were fed the same diets until 8 weeks (average body weight 17 kg). They were randomly divided into 4 groups with each group having two pigs with the same gender and the same parents. For the following 4 weeks, one pig in each group was fed 25 mg/kg clenbuterol twice daily in their diets as the test pig, while the other was fed the same diet without clenbuterol as the control. Then one group of hogs and one group of sows were slaughtered for analysis. These two groups are referred to as the 3 month-old pigs. The other two groups were fed with/without 50 mg/kg clenbuterol twice daily in their diets for another 4 weeks and slaughtered for analysis. These two groups are referred to as the 4 month-old pigs. Approximately 1 g biopsies of different tissues, including the back fat adipose tissues (at the fifth lumbar vertebra level), skeleton muscle (biceps femoris and sural muscle), liver, heart, kidney, spleen and lung, were taken from each pig. Samples were washed in sterile water, snap frozen in liquid nitrogen and stored at -80°C.

### Histology

Tissue samples were fixed in 4% formaldehyde in PBS, embedded in paraffin, and sectioned (5 μm sections). After deparaffinization and rehydration, the sections were washed three times with PBS and stained with H&E.

### RNA preparation, RNA labeling and DNA microarray hybridization

Total RNA of the adipose tissue was extracted with TRIZOL reagent (Invitrogen, Gaithersburg, MD, USA) and further purified with an RNeasy mini kit (Qiagen, Valencia, CA, USA) according to the manufacturers' instructions.

The porcine cDNA microarray was produced at the China Agricultural University. A total of 11520 spots representing 3358 genes (ESTs) were included on the microarray slide. 3358 genes (ESTs) were cloned from the porcine adipose cDNA library and printed in triplicate on each slide. (More details on the specific genes and probe sequences are given by Guo et al. [31]).

A cDNA microarray hybridization analysis was performed with Pronto!™ Universal Microarray Kits (Corning, MA, USA) according to the manufacturers instructions. Briefly, reverse transcription was done with 10 μg total RNA as the template to synthesize cDNA incorporating the fluorescence dyes Cy3-dCTP or Cy5-dCTP. Probes were purified on a Qiagen spin column (Qiagen, Valencia, CA, USA). Dye-labelled cDNA was mixed together with dry dye-labeled cDNA for hybridization (see Pronto Universal hybridization kit Quick Reference Guide).

To avoid dye bias, the experiments were performed in duplicate by dye swap with Cy5-dCTP first used with the test pig and Cy3-dCTP used with the control pig and then Cy3-dCTP used with the test pig and Cy5-dCTP used with the control pig.

Eight cDNA microarray slides were hybridized for the eight pigs.

### DNA microarray Imaging and data analysis

Arrays were scanned with a ScanArray Express scanner (Parckard Bioscience, Kanata, OT, USA) with the obtained images analyzed with GenePix Pro 4.0 (Axon Instruments, Foster City, CA) and Acuity 4.0 (Axon Instruments, Foster City, CA). The resulting microarray data was then normalized using the space and intensity-dependent normalization in the LOWESS program [32]. Each gene was represented in triplicate on each slide. The intensity (median) of each spot was analyzed using the student t-test to identify the differentially expressed genes (P < 0.05). Low quality spots were filtered out before the student t-test analysis. Low quality spots refer to stained spots with bad images or spots with intensities (median) lower than 200 (too weak) or greater than 60000 (saturated). Differentially expressed genes were defined as genes with P values less than 0.05 in the eight microarray slides. The mean ratios of the differentially expressed genes (ESTs) were calculated as the "ratio of the median" of three spots to indicate the trends in the changes of the mRNA abundance.

The microarray data from this research has been deposited in the NCBI Gene Expression Omnibus data repository under accession numbers GSE8093 [33].

### Quantitative Real-Time PCR

Fluorescent real-time PCR was used to confirm the transcriptional differences observed in the microarray results, The real-time PCR was done on an ABI Prism 9700 Sequence Detection system (Applied Biosystems, Foster, CA, USA) using SYBRgreen technology as described by Li et al. [34]. The PCR primer sequences used for the real-time PCR are shown in Table 8. The PCR reaction volume was 20 μl with the following program:

50°C 2 min (activate the uracil-N-glycosylase enzyme);

95°C 10 min (initial denaturation of the cDNA) ;

40 cycles (1 sec at various elevated temperatures for data acquisition): 95°C 20 sec, annealing (according to the specifications for each primer) 20 sec, 72°C 20 sec;

72°C 10 min (reformation of fully duplexed DNA);

The dissolve curve analysis used heating from 65 to 95°C, with increases of 0.2°C per step with the system held 1 sec at each temperature.

All samples were measured in triplicate. Expression was quantified by the relative standard curve method. A standard graph of the cycle threshold (CT) values was obtained from serial dilutions (10^-1^–10^-8 ^copies/well) of Glyceraldehyde-3-phosphate dehydrogenase (GAPDH, a housekeeping gene) cDNA. The quantification was normalized to an endogenous RNA control of the GAPDH or beta2-microglobulin (B2M). An independent sample t-test was used to analyze differences in mRNA expression with/without administration of clenbuterol. Differences were considered to be statistically significant at P < 0.05. Reactions for which the housekeeping gene's CT values were less than 15 or more than 25 were discarded in the calculations because the start cDNA concentration was not appropriate or the cDNA quality was not good enough.

### Proteome analysis

Protein extraction from adipose cells, two-dimensional electrophoresis and in-gel digestion were conducted as described by Lee et al. [35]. After in-gel digestion, samples were dissolved in 4 ml 0.5% aqueous trifluoroacetic acid for mass spectrometric analysis on a Bruker REFLEX III MALDI-TOF-MS (Bruker-Franzen, Bremen, Germany) in positive ion mode at an accelerating voltage of 20 kV with an a-cyano-4-hydroxy cinnamic acid matrix. The resulting peptide mass fingerprint (PMF) was then used in a search of the SWISS-PROT and NCBInr databases using the Mascot search engine [20] with a tolerance of ± 0.2 D and one missed cleavage site.

## Authors' contributions

Jin Zhang participated in the experimental design, animal feeding and sampling, and the microarray creation and hybridization. Jin Zhang also completed the microarray data analysis, real-time PCR experiments and drafted the manuscript. Qiang He conducted the proteomic research. Qiu Y. Liu helped with the real-time PCR for validation of the microarray data. Wei Guo helped with the animal feeding, sampling, microarray creation and hybridization. Wei W Zhang performed the statistical analyses. Xiao X. Hu, Mei X. Deng and Ning Li designed and oversaw the research and assisted in writing the manuscript. All authors read and approved the final manuscript.

**Table 8:** Primers used for the real-time PCR analysis

Genes & clone number	Primer sequence (5' to 3')	PCR product size (bp)	Annealing Temperature
GAPDH	For. ATGGTGAAGGTCGGAGTGA Rev. ATGGGTAGAATCATACTGGA	154 bp	57°C
Beta 2-microglobulin (B2M)	For. TGG TCTTTCTACCTTCTGGCCC Rev. TGTGATGCCGGTTAGTGGTCTC	166 bp	60°C
Apolipoprotein D (rpfat_18926)	For. AGATCCCAGTGAGCTTTGAG Rev. CGTAGTTCTCATAGTCGGTG	233 bp	58°C
PRKAR1A (rpfat_17661)	For. GGCGACGAGGTGCTATCAG Rev. ATGGCATCAAAAATATCAC	159 bp	60°C
COL1A1 (rpfat_8523)	For. TCAAGATGTGCCACTCCGACT Rev. GCCTGTCTCCATGTTGCAGAA	104 bp	60°C
COL1A2 (rpfat_16033)	For. ATATGCACCTTGGACATCGGT Rev. CACGATGCTCTGATCAATCCT	241 bp	60°C
COL3A1 (rpfat_19990)	For. CCTGCTGGAAAGAATGGTGAC Rev. ACGTTCACCGGTTTCACCTT	132 bp	60°C
COL1A2 (rpfat_17393)	For. CCTGGCTCTAGAGGTGAACG Rev. AGCAGGACCAGGATTACCAG	247 bp	60°C
COL3A1 (rpfat_18309)	For. TTTCTTTTATGGCTCCCCCTG Rev. GCGTGTTCGATATTCGAAGAC	101 bp	60°C
SCD (rpfat_16685)	For. AAGGAACTAGAAGGCTGCTC Rev. TGTAGAGCAGCAGCCATCAC	156 bp	58°C
PHPT1 (rpfat_15312)	For. GAAGACACAGTTGAGGACAC Rev. GGACATTGTTCGGAGGATAG	110 bp	60°C
HSL (rpfat_11096)	For. TCCGAATGGAGTCTGCACTGT Rev. CTTCCACTCTGACCTCCAACG	128 bp	60°C
PMP22 (rpfat_18575)	For. CATGAACATTTGCACCACTTG Rev. GTCAGCACCTAATGGTATGGA	133 bp	60°C

For.: Forward

Rev.: Reverse

## Supplementary Material

Additional file 1 Original data of the research. The data include four tables as follow. Table 1. Clenbuterol residue in porcine blood and adipose. Table 2. Body weight of pigs treated with/without clenbuterol. Table 3. Cross-sectional area of muscle fibers of pigs with/without the administration of clenbuterol. Table 4. Number of adipose cells on histological section slides visible through the microscope eyepiece. Click here for file
