# Supplementary material for: Differential gene expression profile in pig adipose tissue treated with/without clenbuterol
Source: BMC Genomics. 2007 Nov 26;8:433. doi: 10.1186/1471-2164-8-433 (PMC2231380; doi:10.1186/1471-2164-8-433)
Supplement: Additional file 1 — Original data of the research. The data include four tables as follow. Table 1. Clenbuterol residue in porcine blood and adipose. Table 2. Body weight of pigs treated with/without clenbuterol. Table 3. Cross-sectional area of muscle fibers of pigs with/without the administration of clenbuterol. Table 4. Number of adipose cells on histological section slides visible through the microscope eyepiece. [file 1471-2164-8-433-S1.pdf]

Additional file 1

Table.1 Clenbuterol residue in porcine blood and adipose

	3 month-old group				4 month-old group			
Pig	Hog 1	Hog 2	Sow 1	Sow 2	Hog 3	Hog 4	Sow 3	Sow 4
Blood (ng/ml)	0.00	10.00	0.00	26.44	0.00	60.57	0.00	176.08
Adipose (ng/ml)	0.00	0.00	0.00	1.62	0.00	5.32	—	—

—: not determined

Table 2. Body weight of pigs treated with/without clenbuterol

	3 month-old group				4 month-old group			
Pig	Hog 1	Hog 2	Sow 1	Sow 2	Hog 3	Hog 4	Sow 3	Sow 4
Clenbuterol (mg/kg BW [a])	0	25	0	25	0	25+50 [b]	0	25+50 [b]
2-month BW (kg)	18.6	17.4	17.5	16.2	16.8	17.2	17.5	18.3
3-month BW (kg)	33 .2	32.6	30.6	29.8	30.5	32	31	32.2
4-month BW (kg)	—	—	—	—	45.6	46.6	46	46.4
Increased BW (kg)	14.6	15.2	13.1	13.6	28.8 (13.7&15.1) [c]	29.4 (14.8&14.6) [c]	28.5 (13.5&15) [c]	28.1 (13.9&14.2) [c]

a. BW : Body weight

b. 25 mg/kg body weight for the 3rd month and 50 mg/kg body weight for the 4th month

c. Increased body weight in the 3rd month and increased body weight in the 4th month

Table 3. Cross-sectional area of muscle fibers of pigs with/without the administration of clenbuterol

	No. 1	No. 2	No. 3	No. 4	No. 5	No. 6	No. 7	No. 8
Test pigs （μm^2）	1976.12	1915.08	1818.08	1593.78	1639.06	1898.21	1793.26	1910.14
Control pigs （μm^2）	1836.02	1917.22	1397.47	1469.39	1499.36	1654.30	1543.33	1764.34

These two groups are significantly different (student t-test p value 0.0244).

Table 4. Number of adipose cells on histological section slides visible through the microscope eyepiece

Eyepiece	No.1	No.2	No.3	No.4	No.5
Number for control pigs	8	10	7	10	8
Number for test pigs	16	24	23	37	28

Amplified 10×40

The cells on upper and left edges were counted only if the cell crossed the edge of the eyepiece.

The two groups are significantly different (student t-test p value 0.0058).
